# Preventing Diseases by Inoculation

**Published:** 1881-07

**Authors:** 


PREVENTING DISEASES BY INOCULATION.—The number of articles and comments upon the subject of preventing or stamping out contagious diseases by inoculations has been, of late, quite considerable. It shows that the remarks made upon the subject in the JOURNAL OF COMPARATIVE MEDICINE were very timely.

The following seems to be about what has been accomplished in this direction so far. The efficacy of inoculation in checking pleuro-pneumonia has been established in Holland, and a prominent veterinary journal in England urges its adoption in that country.

Pasteur claims to be able to prevent chicken cholera by inoculating a modified virus. Burdon-Sanderson and Greenfield, of the Brown Institution, England, claim that they can obtain an efficient "vaccine matter" for splenic fever by cultivating the virus in guinea pigs. Davaine claims similar results by treating the virus in a different way.

Koch and others have succeeded by inoculation in protecting animals against certain septic infections. Grawitz, of Vienna, has succeeded in preventing the toxic effects of certain moulds by previously inoculating the animal with cultivated virus.

There is a suggestive communication on page 193, from Dr. Wesley Miller, of this city, who claims nothing less than the possibility of preventing scarlet fever, and perhaps tuberculosis, by inoculating the cultivated virus of allied diseases existing in the lower animals.

Certainly the field of comparative pathology is rapidly widening. The veterinarian should remember that here he can secure the greatest scientific triumphs. All that is needed is ambition and industry.

